# Anti-amnestic properties of Brahmi and Mandookaparni in a rat model

**DOI:** 10.4103/0019-5545.31554

**Published:** 2006

**Authors:** Chittaranjan Andrade, J. Suresh Chandra

**Affiliations:** * Professor, Department of Psychopharmacology, National Institute of Mental Health and Neurosciences (NIMHANS), Bangalore; ** Officer in Charge, Central Animal Research Facility, NIMHANS

**Keywords:** Electroconvulsive therapy, electroconvulsive shocks, herbal treatments, amnesia, memory, cognition, rats, Brahmi, Mandookaparni

## Abstract

**Background:**

We had previously demonstrated that a complex herbal formulation (Mentat; Himalaya Drug Company, Bangalore) attenuated anterograde and retrograde amnesia induced by electroconvulsive shocks (ECS) in rats. We later showed that a simplified formulation (Memorin; Phyto Pharma, Kolhapur) had similar effects.

**Aims:**

In an attempt to identify the ingredients (of the complex formulation), which purveyed the cognitive benefits, we studied two of the constituent herbs, Brahmi and Mandookaparni, separately and together. The experiments included both active (piracetam) and inactive (vehicle) controls.

**Methods:**

Adult, male, Sprague–Dawley rats (*n*=8 per group) were randomized to receive Brahmi, Mandookaparni, a combination of these two herbs (A300), piracetam, or vehicle from days 1 to 15. On days 11 and 12, the rats were trained in a T-maze using a food-driven paradigm. On days 13 and 14, half the rats in each group received 2 ECS (60 mC charge) per day, 5 hours apart. On day 15, recall of pre-ECS learning was assessed. On day 16, transfer of learning was assessed.

**Results:**

None of the active treatments facilitated pre-ECS learning or influenced ECS seizure duration; however, all showed varying but generally favourable profiles in the attenuation of ECS-induced retrograde and anterograde amnesia. The combination of Brahmi and Mandookaparni showed no especial advantage over the individual herbs.

**Conclusion:**

Brahmi and Mandookparni do not in themselves improve learning; however, each attenuates the amnestic effects of ECS without showing synergism in this beneficial action. Exercises in research and development are indicated to further investigate the anti-amnestic properties of these herbs, and to identify the specific chemical constituents which have procognitive effects.

## INTRODUCTION

Anterograde and retrograde amnesia are important adverse effects of electroconvulsive therapy (ECT). While the attenuation of these cognitive deficits is a subject of much investigation, pharmacological approaches have largely met with limited success.^1^–^3^ During the past decade, we conducted a series of preclinical studies to determine whether herbal medicines available in India could help reduce the cognitive effects of the treatment. We found that Mentat (Himalaya Drug Company, Bangalore), a complex herbal formulation, effectively attenuated both anterograde and retrograde amnestic effects of electroconvulsive shocks (ECS) in different animal models.^4^–^8^ Later, we found that Memorin (Phyto Pharma, Kolhapur), a simplified herbal formulation and roughly a subset of Mentat, had similar effects.^9^,^10^

The logic behind our studies and choice of formulations, and our goals, have been summarized elsewhere.^11^,^12^ In brief, our aim was to identify one among a large choice of herbs which might specifically show efficacy in the attenuation of ECS-induced anterograde and retrograde amnesia; our efforts, therefore, were systematic processes in drug development.

In a previous study,^13^ we found that an aqueous extract of Shankapushpi (*Evolvulus alsinoides*), one candidate herb contained in the Mentat and Memorin formulations, conveyed no cognitive benefits to rats which received ECS. In the present study, we examined the effects of two other herbs: Brahmi (*Bacopa moniera*) and Mandookaparni (*Centella asiatica*). Mentat contains both of these herbs, while Memorin contains only the latter. In Ayurveda, the Indian system of herbal medicine, both Brahmi and Mandookaparni are considered to enhance central nervous system functioning.^14^–^17^

What is known about the activity of these two herbs? In animal models, Brahmi or extracts thereof have been found to sedate, potentiate barbiturate hypnosis, and improve learning on various tasks; glutamatergic mechanisms may be involved.^16^ Ingredients of Brahmi that may be responsible for its cognitive effects include the saponins bacosides A and B.^18^–^20^ Brahmi extracts have been reported to be non-toxic, non-teratogenic and non-mutagenic in rats and monkeys; single and multiple dosing studies in healthy human volunteers have also elicited no adverse effects.^19^

Again in animal models, alcoholic extracts of Mandooka-parni have been found to produce hypothermia, depress activity in a dose-dependent fashion, potentiate barbiturate hypnosis, and exert an anticonvulsant action. Antidepressant activity (possibly mediated through D2 dopamine receptors) and cholinomimetic activity have also been reported. Aqueous extracts have been found to have largely similar effects, except that antidepressant activity is diminished or absent.^16^ An aqueous extract has also been suggested to have nootropic activity.^20^ The herb may act on glycine and cholecystokinin C receptors.^15^

To date, the anti-amnestic efficacy of these two herbs is unknown in the context of ECS. We therefore sought to ascertain whether Brahmi and Mandookarparni attenuate ECS-induced anterograde and retrograde amnesia in a T-maze animal model of learning. We also sought to ascertain whether these two herbs improve learning irrespective of ECS, and whether these herbs influence the ECS seizure duration.

## METHODS

Adult, male, Sprague–Dawley rats weighing 180–220 g at intake were housed 4 per cage with free access to tap water and standard laboratory diet. The rats were maintained in a daylight-driven day–night cycle and were kept in a controlled, disturbance-free environment all through the experiment.

The rats were randomized into five groups (*n*=8 per group). The Brahmi group received an extract of this herb in the dose of 500 mg/kg/day; the total bacoside content was 54.5% in the extract used. The Mandookaparni group received an extract of this herb in the dose of 1250 mg/kg/day; the total triterpenoid content was 20.5% in the extract used. The combined treatment group received A300 in the dose of 800 mg/kg/day; A300 is a formulation containing Brahmi and Mandookaparni in the w/w ratio of 9:21. All extracts and formulations were prepared by hot water extraction (from the respective plants) and drying, and were supplied by Zandu Pharmaceutical Works Limited, Bombay, in accordance with its traditions in Ayurveda. The doses used were based upon preliminary, unpublished, in-house experience of the treatments.

There were both active and inactive control groups; the former received piracetam (Intas Pharmaceuticals Limited, Ahmedabad) in the dose of 200 mg/kg/day while the latter received feed alone. All medications were administered as freshly prepared mixtures with animal feed. To ensure that the medications were completely consumed, the rats were permitted access to food for only 1 hour per day, each evening; after their 23-hour fast, they were temporarily housed one per cage and were provided with a wet food pellet containing the drug in the requisite dose; ad lib feeding for 1 hour was permitted only after the medicated pellet was completely consumed. Ad lib water consumption was permitted throughout the day.

The course of the experiment is outlined in the flow chart (Table 1). From days 1 to 10, the rats received only their respective treatments. On days 11–12, the rats were exposed to the T-maze. Each day, half of the rats in each group were trained to run into the left arm of the maze while the other half were trained to run into the right arm. A food pellet, curtained out of sight at the end of the arm, served as a reward. Rats were alternated between left and right arms so that scent markings and other biases did not influence their movement. On each of these two days of training, the direction of arm learning was kept constant; that is, rats that were trained to run into the left arm were trained to run only to the left, while rats that were trained to run into the right arm were trained to run only to the right.

**Table 1:** Study flow chart

Days 1–10	Treatment with trial drugs
Days 11–12	T-maze training
	Treatment with trial drugs Days
Days 13–14	2 ECS (5 hours apart)
	Treatment with trial drugs
Day 15	Assessment of recall of pre-ECS training
	Treatment with trial drugs
Day 16	Assessment of transfer of learning

On each T-maze training day, trials were continued for each rat until it attained satisfactory learning; this was defined as 9 correct arm entries over 10 consecutive trials. Each rat had two learning scores: the number of trials taken to attain the criterion of satisfactory learning, and the number of wrong arm entries during the period of learning. The procedure followed that described by Bures *et al.,*^21^ as standardized in our laboratory.^11^ Acquisition of learning on days 11–12 was a measure of the extent to which the various treatments facilitated the learning process.

On each of days 13–14, half of the animals in each treatment group received an ECS circa 10 a.m. and again, 5 hours later, circa 3 p.m. The ECS were administered through saline-soaked earclip electrodes at a dose of 60 millicoulombs of charge, using the Niviqure (Bangalore, India; constant current, bidirectional brief-pulse) ECT device. The stimulus comprised 0.8 A, 1.5 msec width, bidirectional square waves at a frequency of 125 pulses per second, administered in a stimulus train 0.4 sec long.

The seizure duration was timed by an experienced observer who used a stopwatch, and was defined as extending FROM the commencement of passage of current TO the termination of convulsive movements, or the onset of asynchronous limb movements, whichever occurred earlier. Rats which did not receive ECS underwent a sham ECS procedure. This was identical to that with ECS, except that no stimulus was administered.

On day 15, the rats were re-exposed to the T-maze to assess the extent to which the ECS disturbed their recall of the pre-ECS learning. The procedure followed was the same as that during the training period, and the direction of arm learning was the same as that maintained pre-ECS. Thus, day 15 scores were a measure of ECS-induced retrograde amnesia, and the degree to which the various treatments were protective in this regard.

On day 16, each rat was trained to run into the opposite arm of the T-maze; the training procedures otherwise remained the same. Since new learning occurred on day 16, the scores of this day were a measure of ECS-induced anterograde amnesia, and the degree to which the various treatments were protective in this regard.

On the days of maze learning and ECS, feeding and the administration of medications were conducted only after the maze trials were completed. This was to ensure that the animals were motivated to perform the food-driven T-maze task.

### Statistical methods

A three-way, mixed-model, repeated-measures analysis of variance was used to ascertain whether the drugs facilitated learning on the two days pre-ECS. In this analysis, the dependant variables were the number of trials required to attain the criterion of satisfactory learning, and the wrong arm entry scores. The between subjects factors were drug (5 levels) and ECS (2 levels), and the within subjects factor was day of assessment (2 levels).

To demonstrate ECS-induced retrograde amnesia and the effect of the treatments thereon, the day 15 learning scores were analysed using a two-way analysis of covariance. The dependant variables were the number of trials and the wrong arm entry scores, and the covariates were the number of trials or wrong arm entry scores (as appropriate) on day 12. Drug and ECS status were the between subjects factors.

To demonstrate ECS-induced anterograde amnesia and the effect of the treatments thereon, the day 16 learning scores were analysed using a two-way analysis of variance. The dependant variables were the number of trials and the wrong arm entry scores. Drug and ECS status were the between subjects factors.

To assess treatment effects on seizure duration, the seizure duration scores were analyzed using a two-way repeated measures multivariate analysis of variance. In this analysis, drug status (2 levels) was the between subjects factor, and occasion of ECS (4 levels) was the within subjects factor.

If the omnibus ANOVA F was significant, post hoc testing was conducted through pairwise comparisons of treatments with vehicle. Alpha for significance was set at 0.05; for the post hoc tests, however, alpha was more conservatively set at 0.02 to offset the risk of a Type 1 error.

## RESULTS

The pre-ECS T-maze learning scores (days 11–12) are presented in Tables 2 and 3. The treatment groups are partitioned in this table to separately show the performances of rats later allocated to true or sham ECS. The analysis of these data shows that, with the solitary exception of the main effect for time, the other main effects, the two-way interactions, and the three-way interaction terms were all non-significant. These results may be interpreted as follows:

**Table 2:** Pre-ECS training results: Number of trails to attain satisfactory learning [data are range and mean (SD); *n*=8/group]

Treatment group	ECS group	Day 11 scores	Day 12 scores
Brahmi	True ECS	10–25	9–11
		13.13 (4.91)	10.25 (0.89)
	Sham ECS	10–21	9–17
		13.88 (3.94)	11.75 (2.60)
Mandookaparni	True ECS	10–15	9–11
		11.88 (1.55)	9.75 (0.71)
	Sham ECS	10–20	9–14
		12.88 (3.31)	10.25 (1.67)
A300	True ECS	11–19	9–17
		14.75 (2.49)	10.25 (2.76)
	Sham ECS	10–17	9–12
		12.75 (2.60)	10.00 (1.07)
Piracetam	True ECS	10–14	9–13
		11.13 (1.46)	10.00 (1.31)
	Sham ECS	9–14	9–11
		11.00 (1.51)	9.75 (0.71)
Vehicle	True ECS	10–15	9–12
		12.25 (1.83)	10.00 (0.93)
	Sham ECS	10–16	9–11
		13.13 (2.42)	9.75 (0.89)
ANOVA results			

Effect	F	df	Significance

Drug	2.27	4,70	NS
ECS	0.18	1,70	NS
Time	71.17	1,70	P<0.001
ECS × drug	0.90	4,70	NS
ECS × time	0.06	1,70	NS
Drug × time	1.77	4,70	NS
ECS × drug × time	0.72	4,70	NS

**Table 3:** Pre-ECS training results: Number of wrong arm entries [data are range and mean (SD); *n*=8/group]

Treatment group	ECS group	Day 11 scores	Day 12 scores
Brahmi	True ECS	1–7	0–2
		2.63 (2.00)	1.25 (0.89)
	Sham ECS	1–6	0–5
		3.13 (1.64)	2.00 (1.51)
Mandookaparni	True ECS	1–4	0–2
		2.38 (0.92)	0.75 (0.71)
	Sham ECS	1–6	0–3
		2.50 (1.60)	1.00 (1.07)
A300	True ECS	2–6	0–3
		3.13 (1.36)	0.63 (1.06)
	Sham ECS	1–4	0–3
		2.25 (0.89)	1.00 (1.07)
Piracetam	True ECS	1–3	0–2
		1.75 (0.89)	0.75 (0.71)
	Sham ECS	0–4	0–2
		1.75 (1.16)	0.75 (0.71)
Vehicle	True ECS	1–6	0–2
		2.38 (1.60)	0.88 (0.64)
	Sham ECS	1–4	0–2
		2.75 (1.16)	0.75 (0.89)
ANOVA results			

Effect	F	df	Significance

Drug	2.28	4,70	NS
ECS	0.42	1,70	NS
Time	87.18	1,70	P<0.001
ECS × drug	0.46	4,70	NS
ECS × time	0.50	1,70	NS
Drug × time	1.02	4,70	NS
ECS × drug × time	0.81	4,70	NS

1. The rats showed significant improvement in learning on day 12 relative to day 11.
2. The various treatments did not facilitate or impair learning.
3. The groups did not differ significantly pre-ECS.

The post-ECS (day 15) recall scores are presented in Table 4. The analysis of these data shows that the main effect for drug, the main effect for ECS, and the ECS x drug interaction were all significant. Along with the results of the post hoc testing, these results may be interpreted as follows:

**Table 4:** Post-ECS (Day 15) recall scores [data are range and mean (SD); *n*=8/group]

Treatment group	ECS group	Number of trails to satisfactory learning	Number of wrong arm entries
Brahmi	True ECS	10–15	1–3
		11.63 (2.07)	1.88 (0.99)
	Sham ECS	9–15	0–2
		10.63 (1.92)	1.13 (0.83)
Mandookaparni	True ECS	9–13	0–3
		11.38 (1.41)	2.00 (1.07)
	Sham ECS	9–12	0–2
		10.00 (1.07)	0.87 (0.83)
A300	True ECS	10–15	1–5
		13.25 (1.75)	2.88 (1.25)
	Sham ECS	9–12	0–3
		10.00 (1.07)	1.00 (1.07)
Piracetam	True ECS	9–13	0–2
		10.88 (1.25)	1.50 (0.76)
	Sham ECS	9–11	0–2
		9.75 (0.89)	0.75 (0.89)
Vehicle	True ECS	12–16	3–5
		13.75 (1.49)	3.38 (0.74)
	Sham ECS	9–11	0–2
		9.63 (0.74)	0.75 (0.71)
ANOVA results: Number of trials to satisfactory learning

Effect	F	df	Significance

Drug	3.21	4,69	p=0.018
ECS	57.06	1,69	p<0.001
Drug × ECS	3.61	4,69	p=0.01
Post hoc comparisons: Brahmi vs placebo, p=0.053; Mandookaparni vs placebo, p=0.006; A300 vs placebo, p=0.33; piracetam vs placebo, p=0.001			
ANOVA results: Number of wrong arm entries			

Effect	F	df	Significance

Drug	3.00	4,69	p=0.024
ECS	50.52	1,69	p<0.001
Drug × ECS	2.89	4,69	p=0.028
Post hoc comparisons: Brahmi vs placebo, p=0.008; Mandookaparni vs placebo, p=0.026; A300 vs placebo, p=0.36; piracetam vs placebo, p=0.002

1. ECS produced significant retrograde amnesia.
2. Brahmi, Mandookaparni, and piracetam, but not A300, significantly attenuated measures of ECS-induced retro-grade amnesia.

The post-ECS (day 16) new learning scores are presented in Table 5. The analysis of these data shows that the main effect for drug was not significant; the main effect for ECS and the ECS x drug interaction were, however, both significant. Along with the results of the post hoc testing, these results may be interpreted as follows:

**Table 5:** Post-ECS (Day 16) recall scores [data are range and mean (SD); *n*=8/group]

Treatment group	ECS group	Number of trails to satisfactory learning	Number of wrong arm entries
Brahmi	True ECS	10–13	1–3
		11.25 (1.04)	2.13 (0.83)
	Sham ECS	10–13	1–4
		10.88 (1.25)	1.87 (1.25)
Mandookaparni	True ECS	10–14	1–3
		12.00 (1.41)	2.38 (1.06)
	Sham ECS	10–13	1–3
		11.00 (0.93)	1.88 (0.64)
A300	True ECS	10–14	1–4
		11.88 (1.36)	2.50 (1.07)
	Sham ECS	11–13	2–3
		11.38 (0.74)	2.13 (0.35)
Piracetam	True ECS	10–16	1–4
		12.00 (1.77)	2.63 (0.92)
	Sham ECS	9–15	0–4
		11.38 (1.85)	2.00 (1.31)
Vehicle	True ECS	11–18	2–7
		14.00 (2.62)	3.88 (1.73)
	Sham ECS	10–11	1–2
		10.63 (0.52)	1.63 (0.52)
ANOVA results: Number of trials to satisfactory learning			

Effect	F	df	Significance

Drug	1.50	4,70	NS
ECS	12.81	1,70	p<0.001
Drug × ECS	2.91	4,70	p=0.028
Post hoc comparisons: Brahmi vs placebo, p=0.011; Mandookaparni vs placebo, p=0.042; A300 vs placebo, p=0.014; Piracetam vs placebo, p=0.045			
ANOVA results: Number of wrong arm entries			

Effect	F	df	Significance

Drug	1.19	4,70	NS
ECS	11.79	1,70	p<0.001
Drug × ECS	2.91	4,70	p=0.05
Post hoc comparisons: Brahmi vs placebo, p=0.023; Mandookaparni vs placebo, p=0.032; A300 vs placebo, p=0.019; Piracetam vs placebo, p=0.067

1. ECS induced significant anterograde amnesia.
2. Brahmi and A300 significantly attenuated measures of ECS-induced anterograde amnesia; while anterograde amnesia was less with Mandookaparni and piracetam as compared with vehicle, the effects just failed to reach statistical significance.

The seizure duration data are presented in Table 6. The analysis of these data showed that seizure duration decreased significantly across occasions of ECS, and that seizure duration did not vary as a function of treatment.

**Table 6:** Seizure duration (secs) in ECS-treated rats [data are range and mean (SD); *n*=8/group]

Treatment group	Day 13 ECS 1	Day 13 ECS 2	Day 14 ECS 3	Day 14 ECS 4
Brahmi	10–18	11–20	8–13	10–14
	13.88	13.88	10.75	12.25
	(3.00)	(2.90)	(2.25)	(1.91)
Mandookaparni	10–18	10–17	9–17	9–16
	13.50	13.38	11.63	12.88
	(2.93)	(2.20)	(2.88)	(2.75)
A300	10–20	10–18	8–15	6–20
	13.25	13.75	11.63	12.25
	(3.24)	(2.82)	(2.50)	(4.03)
Piracetam	10–18	10–16	9–16	6–17
	14.25	13.25	13.00	12.38
	(2.55)	(2.25)	(2.56)	(3.85)
Vehicle	10–17	10–15	9–15	9–16
	12.63	13.00	12.88	12.25
	(2.83)	(1.77)	(2.23)	(2.38)
RMANOVA results				
Main effect for drug: F=0.08, df=4,35, NS				
Main effect for time: Pillai's criterion=0.49, F=10.46, df=3,33, p<0.001				
Drug × time: Pillai's criterion=0.36, F=1.18, df=12,105, NS				

## DISCUSSION

Both Brahmi and Mandookaparni have been reported to have sedating effects.^16^ Such effects can impair performances on memory tasks that require significant motor activity.^12^ While we did not study the effects of the treatments on motor activity, we do not believe that sedation, if present with the extracts and at the doses used in our study, was a confounding variable. Had sedation indeed been a source of bias, learning performances would have been impaired, and not improved as we observed (Tables 4, 5). Furthermore, sedation generally biases the results of tasks that depend upon timings (e.g. conditioned avoidance paradigms) rather than those which depend upon choices (e.g. T-maze paradigms).

In one regard, our observations were incongruent with those reported in literature. Mandookaparni has been suggested to delay pentylenetetrazol-induced seizures in mice;^16^ yet, we found that the herb did not interfere with the ECS seizure duration (Table 6). Differences in the experimental models, and in the dependent variables, are likely to be responsible for the dissimilarity of the findings.

We found that none of the active treatments enhanced pre-ECS learning. This suggests that the treatments do not have an independent, beneficial effect on memory processes. However, it is possible that with a larger sample size, or a longer duration of pre-ECS treatment administration, or a more difficult learning task, advantages for the treatments may have been obtained.

Both herbs, and their combination, showed varying but generally favourable profiles of anti-amnestic action in the context of ECS. The mechanism of anti-amnestic action of these herbs is unknown. While longer ECT seizures may be associated with greater cognitive deficits,^22^ the herbs did not appear to act through an interference with the ECS seizure, at least insofar as seizure duration was concerned (Table 6). The cognitive benefits associated with these herbs confirm Ayurvedic beliefs^17^ and the results obtained with other animal models.^14^,^18^–^20^

In Ayurvedic medical practice, combination therapy is the rule rather than the exception; the underlying expectation is that the herbs in the combination will augment each other in efficacy and diminish each other in adverse effects. While it may be argued that the proportion of the herbs in the A300 formulation (Brahmi:Mandookaparni=9:21) may not necessarily have been optimum, the absence of therapeutic synergism with these herbs was striking: the combination did not attenuate ECS-induced retrograde amnesia (Table 4), and was little better than the individual herbs in decreasing ECS-induced anterograde amnesia (Table 5). The lack of synergism suggests at the very least that the mechanism of action of these two herbs may involve a final common pathway; the absence of benefit in the retrograde amnesia model, in fact, raises a needle of suspicion that antagonistic mechanisms may be at work. Whether or not the combination reduces adverse effects is hard to determine from animal experiments.

We conclude that Brahmi and Mandookaparni both merit further investigation in amnestic contexts. It is conceivable that a further analysis of these herbs may lead to the isolation of one or more active principles which have precognitive action.
